# Serum Pepsinogens Combined with New Biomarkers Testing Using Chemiluminescent Enzyme Immunoassay for Non-Invasive Diagnosis of Atrophic Gastritis: A Prospective, Multicenter Study

**DOI:** 10.3390/diagnostics12030695

**Published:** 2022-03-12

**Authors:** Nicolas Chapelle, Malgorzata Osmola, Jérôme Martin, Justine Blin, Maxime Leroy, Iva Jirka, Driffa Moussata, Dominique Lamarque, Raphael Olivier, David Tougeron, Anne Hay-Lombardie, Edith Bigot-Corbel, Damien Masson, Jean-François Mosnier, Tamara Matysiak-Budnik

**Affiliations:** 1 IMAD, Hepato-Gastroenterology & Digestive Oncology, University Hospital of Nantes, Hôtel Dieu, Place Alexis Ricordeau, CEDEX 1, 44093 Nantes, France; nicolas.chapelle@chu-nantes.fr (N.C.); iva.jirka@chu-nantes.fr (I.J.); 2 INSERM U1064 CRTI, 44093 Nantes, France; jerome.martin@univ-nantes.fr; 3 Faculty of Medicine, University of Nantes, 44300 Nantes, France; justine.blin@chu-nantes.fr (J.B.); edith.bigot@chu-nantes.fr (E.B.-C.); damien.masson@chu-nantes.fr (D.M.); jeanfrancois.mosnier@chu-nantes.fr (J.-F.M.); 4 Department of Immunology, University Hospital of Nantes, 44093 Nantes, France; mal.osmola@gmail.com; 5 Department of Hematology, Transplantation and Internal Medicine, Medical University, 02-091 Warsaw, Poland; 6 Department of Biochemistry, University Hospital of Nantes, 44093 Nantes, France; anne.haylombardie@chu-nantes.fr; 7 INSERM U1235 TENS, 44300 Nantes, France; 8 Department of Biostatistics, CHU de Nantes, 44093 Nantes, France; maxime.leroy@chu-nantes.fr; 9 Department of Hepato-Gastroenterology, University Hospital of Tours, 37044 Tours, France; d.moussata@chu-tours.fr; 10 Department of Hepato-Gastroenterology, Ambroise-Paré Hospital, AP-HP, Paris Saclay University, UVSQ, INSERM, Infection and Inflammation, 91190 Paris, France; dominique.lamarque@aphp.fr; 11 Department of Hepato-Gastroenterology, Poitiers University Hospital, University of Poitiers, 86000 Poitiers, France; raphael.olivier@chu-poitiers.fr (R.O.); david.tougeron@chu-poitiers.fr (D.T.); 12 Department of Pathology, University Hospital of Nantes, 44093 Nantes, France

**Keywords:** atrophic gastritis, non-invasive markers, pepsinogens, diagnostic performance

## Abstract

Background: Analysis of serum biomarkers for the assessment of atrophic gastritis (AG), a gastric precancerous lesion, is of growing interest for identification of patients at increased risk of gastric cancer. The aim was to analyze the diagnostic performance of serum pepsinogen testing using another method, chemiluminescent enzyme immunoassay (CLEIA), as well as of other new potential biomarkers. Material and Methods: The sera of patients considered at increased risk of gastric cancer and undergoing upper endoscopy collected in our previous prospective, multicenter study were tested for pepsinogen I (PGI) and II (PGII), interleukin-6 (IL-6), human epididymal protein 4 (HE-4), adiponectin, ferritin and Krebs von den Lungen (KL-6) using the CLEIA. The diagnostic performance for the detection of AG was calculated by taking histology as the reference. Results: In total, 356 patients (162 men (46%); mean age 58.6 (±14.2) years), including 152 with AG, were included. For the detection of moderate to severe corpus AG, sensitivity and specificity of the pepsinogen I/II ratio were of 75.0% (95%CI 57.8–87.9) and 92.6% (88.2–95.8), respectively. For the detection of moderate to severe antrum AG, sensitivity of IL-6 was of 72.2% (95%CI 46.5–90.3). Combination of pepsinogen I/II ratio or HE-4 showed a sensitivity of 85.2% (95%CI 72.9–93.4) for the detection of moderate to severe AG at any location. Conclusion: This study shows that PG testing by CLEIA represents an accurate assay for the detection of corpus AG. Additionally, IL-6 and HE-4 may be of interest for the detection of antrum AG. Mini-abstract: Pepsinogens testing by chemiluminescent enzyme immunoassay is accurate for the detection of corpus atrophic gastritis. IL-6 and HE-4 maybe of interest for the detection of antrum atrophic gastritis.

## 1. Introduction

Gastric cancer (GC) incidence has been decreasing over the past five decades in parallel to the decreasing prevalence of *H. pylori* infection [1]. However, it still represents the fifth most common cancer and the third leading cause of cancer-related death in the world. GC incidence varies considerably among different countries, being particularly high in the “Eastern world” (annual incidence rates up to 60/100,000 in East Asia) as compared with the “Western world” (annual incidence rates varying from 5/100,000 to 10/100,000 in Western Europe or USA) [2]. France is classically described as a low-risk GC area, with incidence rates around 7/100,000 in males and 2.6/100,000 in females [3].

Although important progress has been made in the field of cancer treatment, the overall survival in GC remains poor and is closely related to the stage of the disease at diagnosis [4]. Thus, as in other cancers, making early diagnosis is the best way to improve prognosis in GC. For decades, the Correa cascade of gastric precancerous lesions (GPL)—i.e., atrophic gastritis (AG), intestinal metaplasia (IM), low grade dysplasia (LGD), and high grade dysplasia (HGD), appearing successively following chronic infection with *H. pylori*—has been described and considered as the main pathway of gastric carcinogenesis [5,6]. Large population-based studies demonstrated increasing risk of GC parallel to the increasing severity of the lesions [7,8], and most of the studies on GPL focused on AG and IM, which are the most commonly observed [9,10,11]. In Asia, the knowledge of gastric physiology and carcinogenesis has led to the development of blood tests, and especially pepsinogen testing, which have shown their usefulness for the stratification of the patients according to their GC risk (“ABC method”) [12]. In Western countries, the standard method of assessing the status of the gastric mucosa remains histological analysis of gastric biopsies obtained during an upper endoscopy, which is an invasive, costly, and often not well-accepted procedure. Moreover, the correlation between endoscopic evaluation of the mucosa and histologic findings is very poor [9], and there is a risk of false diagnosis due to the sampling error since the distribution of the GPL may be patchy. 

However, the recent European guidelines recognize the usefulness of pepsinogen testing for identifying the most at-risk patients in whom endoscopic evaluation would be required [13]. Pepsinogen I (PGI) is secreted by the chief cells present only in the corpus mucosa, while pepsinogen II (PGII), is secreted by both antrum and corpus cells. The decrease in PGI level and in the PGI/PGII ratio is considered a marker of gastric, and especially corpus, atrophy. Combination of biomarkers, as proposed in the Gastropanel^®^ (PGI, PG II, Gastrin 17: G-17, and *H. pylori* serology), based on enzyme linked immunosorbent assay (ELISA), has shown promising results for the diagnosis of AG [14], although wide variations in its diagnostic accuracy among the different populations studied have been observed [15]. We have previously reported the results of Gastropanel^®^ in France [16], which has shown good diagnostic performance for the detection of corpus AG and of severe atrophy, but which has been insufficient for the detection of antral or mild atrophy. In the present study, we wanted to evaluate in the same setting another method for pepsinogen testing, ChemiLuminescent Enzyme ImmunoAssay (CLEIA), which has never been used for the detection of gastric atrophy in a European population. Our second aim was to test other potential biomarkers, i.e., adiponectin, human epididymal protein 4 (HE-4), interleukin-6 (IL-6), Krebs von den Lungen 6 (KL-6) and ferritin, which according to some published data could be involved in gastric carcinogenesis [17,18,19,20]. We hypothesized that blood level of these markers could be increased in GPL, and in consequence, they could increase our ability to detect gastric atrophy, and in particular antrum atrophy, for which no validated markers exist.

## 2. Patients and Methods

### 2.1. Design of the Study

This study was based on the analysis of the sera collected during our previous prospective, multicenter study, including all the consecutive patients considered at increased risk for GC, presented between 2016 and 2019 in four French University Hospitals for an upper endoscopy with gastric biopsies. The sera collected during that study were kept frozen, until being retrieved for the present analysis. The details on patients’ selection, endoscopy protocol used, blood sample collection, and histological evaluation of gastric biopsies are described in our previous article [16]. Briefly, all the consecutive patients considered at risk for GC were proposed for inclusion. An upper endoscopy with at least 4 gastric biopsies (2 from the antrum and 2 from the corpus) was performed and a fasting blood sample was obtained. The presence, intensity, and distribution of GPL (AG and IM) were evaluated using the updated Sydney system [21]. According to the results of histopathological analysis, the patients were classified into 5 groups: normal gastric mucosa (N), non-atrophic gastritis (NAG), AG restricted to the antrum (AGA), AG restricted to the corpus (AGC), and AG extended to the antrum and to the corpus (AGAC). Additionally, patients with moderate to severe AG were distinguished from the patients with mild AG. 

### 2.2. Measurement of Serum Biomarkers

Serum biomarkers (HE4, IL6, KL6, Adiponectin, Pepsinogen I and II) were analyzed using the CLEIA (ChemiLuminescent Enzyme ImmunoAssay) on the fully automated LUMIPULSE G instrument (Fujirebio^®^ France SARL, Courtaboeuf, France). The system uses a unique mono test cartridge concept for the quantitative determination of each parameter. Ferritin was analyzed by immunoturbidimetric method (Cobas 8000, Roche^®^, Basel, Switzerland).

### 2.3. Statistical Analysis

The diagnostic accuracy of the markers was assessed by receiver operating characteristic (ROC) curve analysis, with evaluation of sensitivity (Se), specificity (Sp), positive predictive value (PPV), and negative predictive value (NPV). Because of the selection of the patients for this study, to better explore the performance of the test independent of the prevalence of GPL in the studied population, in addition to PPV and NPV, the positive likelihood ratio (PLR) and the negative likelihood ratio (NLR) were calculated. Statistical analysis was performed separately for AG of the antrum, of the corpus, and of the whole stomach, as well as according to the severity of AG (graded as mild or moderate/severe). 

For pepsinogens, the cut-off levels commonly recommended in the Western populations (PGI: <30 μg/L; PGII: <3 μg/L, PGI/PGII ratio: <3.0) were used, and the values below these cut-off levels were considered as indicators of atrophy. Additionally, the ROC curves were developed to establish the best cut-off values for the study population using CLEIA technique (Youden’s index). For other markers, since no recommended cut-off values are available, the evaluation was based on the best cut-off values identified by the ROC curves analysis for each parameter. 

The ANOVA and post hoc Tukey test analysis were used to compare the values obtained for different biomarkers, considered alone or in combination, by taking histology as the reference. Statistical analysis was performed using the R version 3.6.0. software.

## 3. Results

### 3.1. Patients—Serum Samples

From the 397 serum samples initially collected, 7 were excluded from the initial study (5 because of synchronous adenocarcinoma and 2 for not fulfilling the inclusion criteria), 29 were not analyzed because of an incomplete biopsy protocol, and 5 others were not available. Finally, 356 patients (162 men (46%); mean age 58.6 (±14.2) years) were included in the study. Mean age in N, NAG, AGA, AGC, and AGAC groups were 56.1 (±14.3), 56.9 (±14.1), 61.9 (±12.2), 62.6(±14.2), respectively. The mean delay between endoscopy and blood sample intake was 5.4 days (Q1:0.0; Q3: 0.0), and 79% of blood samples were collected the day of endoscopy. 

### 3.2. Histology 

According to the results of histopathological analysis, the patients were categorized into three groups: those with a normal gastric mucosa (N) (*n* = 113, 48 males, mean age 56.1 (±14.3) years), those with a non-atrophic gastritis (NAG) (*n* = 91, 37 males, mean age 56.9 (±14.1) years), and those with AG (*n* = 152, 77 males, mean age 61.4 (±13.8) years). Furthermore, within the group of the patients with AG, three groups were distinguished: patients with antrum-limited AG (AGA) (*n* = 72), corpus-limited AG (AGC) (*n* = 42), and pangastric (involving antrum and corpus) AG (AGAC) (*n* = 38).

In 129 out of 152 patients with AG (84.0%), IM was also present. *H. pylori* infection was found in 47 out of 356 patients (13.2%) by histology and in 61 patients (17%) by serology. Advanced gastric atrophy or IM (graded as moderate or severe) according to the Sydney classification was found in 54 out of 152 patients (35.5%).

### 3.3. Serum Biomarkers Testing Results

The results of the tests are presented according to the clinical situations of interest encountered by the clinicians—i.e., AG restricted to the antrum (AGA), to the corpus (AGC) or extensive, pangastric AG (AGAC). Additionally, the results for the patients with the most severe lesions (moderate or severe atrophy) are presented since the patients harboring these lesions are considered at the highest risk of progression to cancer. Because the patients with non-atrophic gastritis (NAG) are not considered at increased risk for GC, in some analyses they were categorized together with the patients with normal gastric mucosa (N) as controls. However, separate results for these two categories of patients, for each marker, and for each clinical situation are available upon request. Since PPI-therapy may influence the results of certain markers (particularly pepsinogens), the results for long-term PPI users were analyzed separately. 

The values of all biomarkers studied, PG I, PGII, PG/PGII ratio, adiponectin, ferritin, HE-4, IL-6, and KL-6, according to different histological groups, are presented in Table 1. Post hoc analysis (Tukey’s) for 2 by 2 comparison is available in Supplementary Table S1.

Pepsinogens

Patients with AGC had significantly decreased PGI levels as compared with N (*p* < 0.001), NAG (*p* < 0.001), and AGA (*p* < 0.001) patients, and borderline as compared with AGAC patients (*p* = 0.051). For PGII, the difference was statistically significant only between AGC and AGA patients (*p* = 0.039). PGI/PGII ratio was significantly lower in patients with AGC than in patients with N (*p* < 0.001), NAG (*p* < 0.001), AGA (*p* < 0.001), and AGAC (*p* < 0.001). Similarly, the PGI/PGII ratio was significantly lower in patients with extensive AG (AGAC) as compared with N (*p* < 0.001), NAG (*p* = 0.001), and AGA (*p* = 0.004) patients. There was no significant difference in PG levels between the AGA patients and N (*p* = 0.756) or NAG (*p* = 0.999) patients (Supplementary Table S1). 

HE-4 

A significantly higher level of HE-4 was found in patients with AGAC as compared with N (*p* = 0.020) and NAG (*p* = 0.011) patients. 

Other markers

No significant difference was found among the different groups for adiponectin, ferritin, IL-6, or KL-6 (Table 1). 

(1) Diagnostic performance of biomarkers for the detection of any atrophy (AGA, or AGC, or AGAC) 

For the detection of any gastric atrophy, PGI/PGII ratio showed the best performance, with Se and Sp of 44.7% (95%CI 36.7; 53.0) and 92.6% (95%CI 88.2; 95.8), respectively, using a standard cut-off <3.0 (AUC 0.685). The corresponding values for the best cut-off (<3.03) were of 46.7% (95%CI 38.6; 55.0) and 92.6% (95%CI 88.2; 95.8), respectively. This performance was improved in the case of moderate to severe atrophy, with Se of 57.4% and Sp of 92.6% for the best cut-off (Table 2).

**Figure 1:**
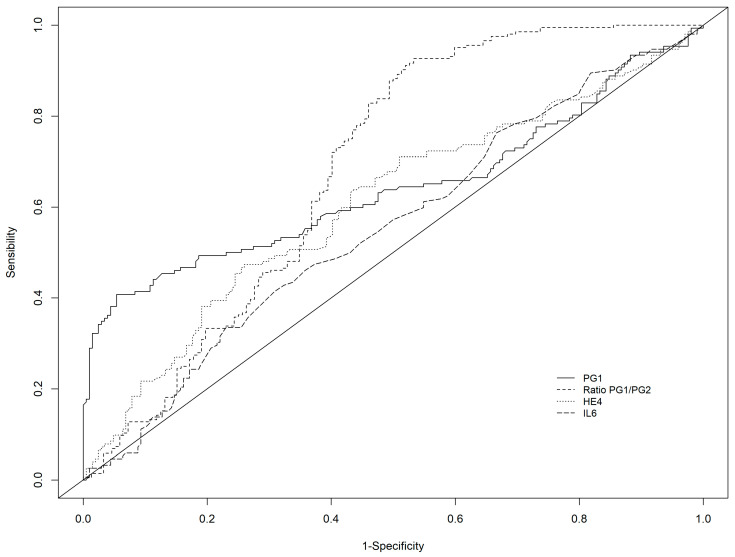
Receiver operating characteristic curve of PGI, PGI/PGII ratio, HE-4, and IL-6 for the detection of any atrophy (AGA or AGC or AGAC).

Among other markers, the best diagnostic performance was observed with HE4, in particular in combination with PGI/PGII: Se of 69.7% and Sp 67.6% with AUC of 0.687 for any atrophy and Se of 85.2% and Sp of 52.0% with AUC 0.686 for moderate to severe atrophy (Table 2, Figure 1). 

Associations of biomarkers allowed an increase in Sp or Se, whether they were used together (marker 1 AND marker 2) or independently (marker 1 OR marker 2). To maximize Se, the most interesting combination for the detection of any AG was PGI/PGII OR HE4, with Se of 69.7% (95%CI 61.8–76.9) and Sp of 67.6% (95%CI 60.8–74.0) (cut-off: PGI/PGII <3.03, HE4 >75.8 µg/mL) for the detection of any AG. To maximize Sp, the best combination of biomarkers for the detection of any AG was the association of PGI/PGII and HE4, giving a Sp of 99.0% (95%CI 96.5–99.9) but a Se of only 23.7% (95%CI 17.2–31.3). 

(2) Diagnostic performance for the detection of corpus atrophy 

**Figure 2:**
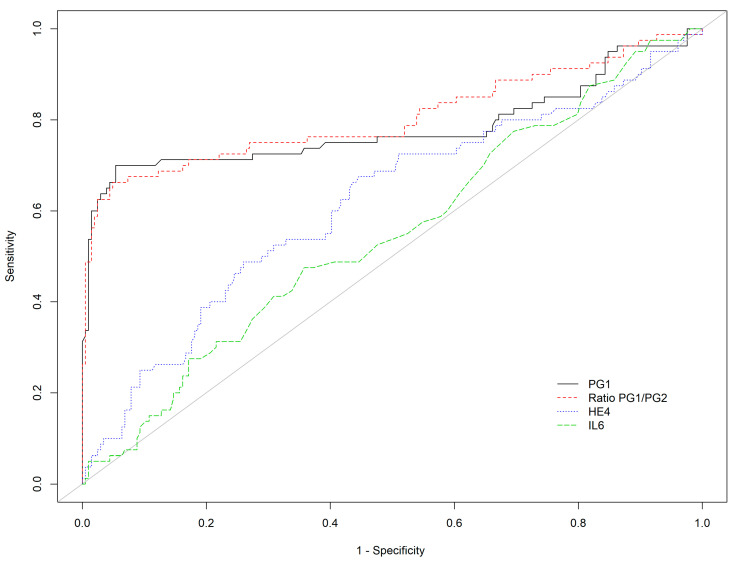
Receiver operating characteristic curve of PGI, PGI/PGII ratio, HE-4, and IL-6 for the detection of corpus AG (AGC + AGAC).

With the commonly used cut-off (<30 μg/L), PG I showed a Se of 71.2% and Sp of 83.8% for the detection of corpus AG, with corresponding PLR and NLR values of 4.4 and 0.34, respectively. Results were comparable for PGI/PGII ratio, with Se of 67.5% and Sp of 92.6% (PLR and NLR of 9.18 and 0.35, respectively). The results were improved in the case of moderate to severe corpus AG (PGI: Se 77.8%, Sp 83.8%; PGI/PGII: Se 75.0%, Sp 92.6%). PGI and PGI/II were superior to all other markers for the detection of AGC. (Table 3, Figure 2). 

(3) Diagnostic performance for the detection of antrum atrophy 

**Figure 3:**
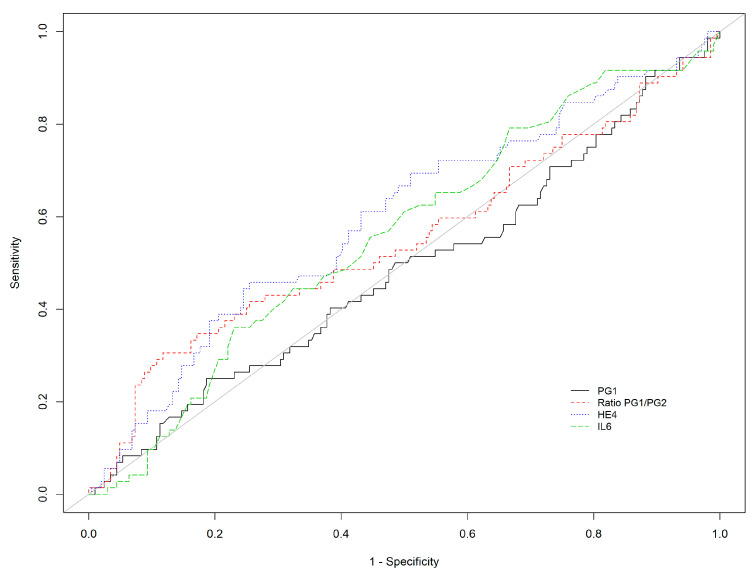
Receiver operating characteristic curve of PGI, PGI/PGII ratio, HE-4, and IL-6 for the detection of antrum AG (AGA).

As expected, pepsinogens were not efficient for the detection of AGA, and the results are not provided in Table 4 (but can be available upon request) since the PGI levels of the patients with AGA were even slightly above the level of control patients (N + NAG). Among the other markers, HE4 and IL-6 yielded the bests results, with Se of 66.7% (95%CI 41.0–86.7) and 72.2% (95%CI 46.5–90.3), respectively, for the detection of moderate to severe antrum atrophy. Surprisingly, adiponectin showed a Se of 58.3% for the detection of any antrum AG but only of 22.2% for the detection of moderate to severe AG. KL6 showed a very good Se (77.8%) for the detection of antrum AG, especially severe AG (94.4%), but with a very poor Sp (Table 4, Figure 3).

(4) Diagnostic performance for the detection of the pangastric (antrum and corpus) atrophy 

**Figure 4:**
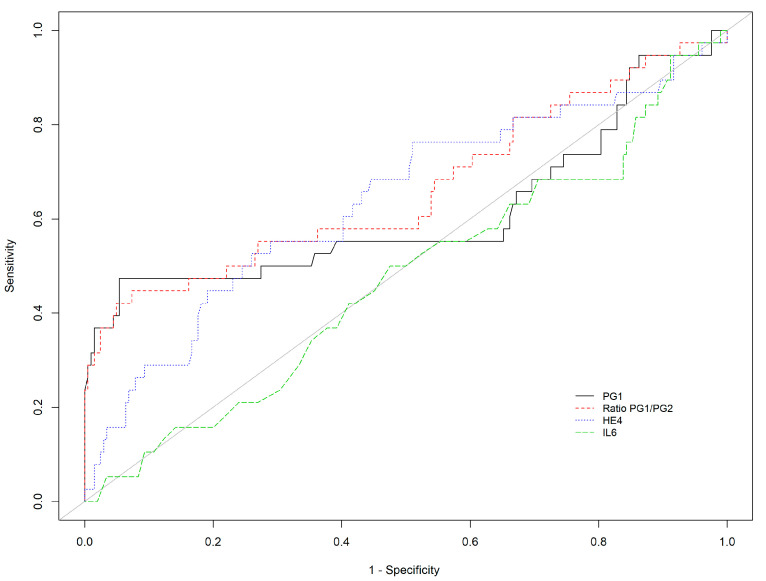
Receiver operating characteristic curve of PGI, PGI/PGII ratio, HE-4, and IL-6 for the detection of extensive AG (AGAC).

Among all the biomarkers tested, PGI/PGII ratio (cut-off <3) and HE4 (cut-off >75.8 µg/mL) showed the best performance for the detection of pangastric atrophy, with an AUC of 0.664 and 0.638 and Se of 44.7% (95%CI 28.6–61.7) and 52.6% (95%CI 35.8–69), respectively (Table 5, Figure 4). 

(5) Diagnostic performance for the detection of moderate to severe atrophy

The diagnostic performance of PG and HE-4 increased in the case of moderate to severe atrophy as compared with any atrophy: Se and Sp for PGI/PGII ratio (cut off <3.03) were of 57.4% and 92.6%, for PGI/PGII ratio, respectively, and for HE4 (cut off >63.2 µg/mL) of 70.4% and 55.4%, respectively (Table 2). Corresponding AUCs for PGI/PGII ratio and HE4 were 0.740 and 0.637, respectively (Figure 2). A combination of markers allowed a further increase in Se up to 85.2% (95%CI 72.9–93.4). Consequently, the most interesting NLR for the detection of moderate to severe atrophy was obtained with a combination of PGI/PGII (<3.03) or HE4 (>63.2 µg/mL): 0.29 (95%CI 0.15–0.55). The best PLR was obtained with PGI/PGII ratio (7.56 (95%CI 4.39; 13)) (Table 2). 

### 3.4. Diagnostic Performance in Patients without PPI Therapy

There was no significant change when analyzing the performance of the markers in this subgroup of patients (Supplementary Table S2). 

### 3.5. Comparison between H. pylori-Positive and H. pylori-Negative Patients

There was no significant difference in PGI level (Mean ± SD) between *H. pylori*-positive patients (56.44 ± 42.91 ng/mL) and *H. pylori*-negative patients (59.99 ± 62.09 ng/mL, *p* = 0.594). However, *H. pylori*-positive patients, presented a lower PGI/PGII ratio (3.40 ± 1.58) than *H. pylori*-negative patients (4.30 ± 2.19, *p* < 0.001), and this difference was particularly observed in the group of control patients (3.69 ± 1.27 vs. 4.92 ± 1.55, respectively, *p* < 0.001), while it was not statistically significant in the group of the patients with AG (3.18 ± 1.78 vs. 3.37 ± 2.65, respectively, *p* = 0.619). Consequently, the PGII level was significantly higher in *H. pylori*-positive patients (17.34 ± 10.01) than in *H. pylori*-negative patients (13.34 ± 11.64, *p* = 0.007).

### 3.6. Comparison between the Results of the Previous Study (Gastropanel^®^) and the Current Study (CLEIA Fujirebio^®^)

There was not a significant difference in the diagnostic performance for the detection of any atrophy or corpus atrophy between the two tests, either for PGI or for PGI/PGII ratio (Supplementary Table S3).

## 4. Discussion

Our study is to our knowledge the first report of pepsinogen testing for the detection of AG using CLEIA in Europe. Only two studies have tested this technique so far, both of them performed in Japan, with one showing the normalization of PG levels after eradication of *H. pylori* [22] and the other showing that PG testing may be useful in classifying GC risk according to ABCD classification [23]. 

The PG I and PGII, whose levels reflect the functional state of the gastric mucosa, are the most validated markers. We report here a good performance of PGI and PGI/PGII ratio measured by CLEIA for the detection of corpus AG, with a Se of 70% and Sp of over 94%. The sensitivity of this test further increases in the case of severe AG (about 78%), indicating that the more the atrophic lesions are pronounced, the more sensitive is the test. This observation is important from clinical point of view since the patients with more severe lesions are considered at most at risk of gastric cancer. These results are comparable with those achieved in most of the studies reported in the literature [24] and similar to those obtained in the same population in our previous study using ELISA assay [16]. Thus, this study shows that CLEIA is not only technically easy (results available in 20 min) but also efficient for the detection of corpus AG. Indeed, this technique is of growing interest in biology laboratories due to its easy use in a routine practice [25]. In a previous publication by Leja and colleagues, the comparison of three assays (two of them using ELISA and one using a latex agglutination test) did not show any significant changes in the diagnostic performance of pepsinogens among the different techniques used [26]. 

One of the weaknesses of non-invasive diagnosis of AG using PG testing is its relative low level of performance for the diagnosis of antrum atrophy. Although current evidence suggests that corpus atrophy is a major marker of risk of progression to GC, several studies have demonstrated that the most common location of gastric atrophy is the antrum [9,10,11,27] and that not only the location but also other parameters, such as severity of atrophy or incomplete type of intestinal metaplasia, are important factors associated with an increased risk of GC [28,29,30]. There is no currently established marker for the detection of antral atrophy. Some previous studies evaluated the diagnostic value of gastrin in this indication, but the results were discordant, and there were important methodological issues that made this marker less useful in clinical practice [31]. Therefore, we tried to investigate other potential markers of impaired gastric function in addition to pepsinogens—namely, those that have been reported to be involved in gastric carcinogenesis, particularly in the development of IM, and those whose value in the detection of GPL has not been investigated yet. 

Adiponectin is a hormone whose blood concentration is inversely correlated with the level of visceral abdominal fat, and which has been associated with various human diseases [32]. It is believed to play a role in several malignancies through various mechanisms, among which are the regulation of cytokines and hormone release, insulin-resistance, and tumor cell proliferation [17]. A low adiponectin level has been associated with an increased risk of GC and has been correlated with clinical stage [33]. In our study, with a Se of 58%, serum adiponectin did not appear as a marker with performance sufficiently high to be considered as a potential candidate marker for the detection of AG. Krebs von Lungen 6, which is a subtype of mucin 1 (MUC1), has been mostly investigated in biliary or pancreatic cancers [18]. However, several studies have also shown aberrant expression of MUC1 in GC, which could be associated with deeper invasion and lymph node metastasis [18,34]. Although in our study, KL6 showed a very good Se for the detection of antral atrophy, and especially severe atrophy (>90%), due to a very low Se (22.5%), this marker does not appear reliable as a detection marker. 

It has been suggested that consecutively to *H. pylori* infection and inflammation, the IL-6/STAT3 signaling pathway is activated, promoting epithelial to mesenchymal transition [19]. Increased levels of IL-6 and other chemokines have been associated with GC growth, and IL-6 serum level has been shown to increase in parallel to tumor progression and to be correlated with survival. Several studies have investigated the IL-6 value as a diagnostic marker of established GC, with a wide range of Se and Sp reported (0.39–0.85 and 0.50–0.97, respectively) and a wide variation in the cut-off values used [35,36,37]. In our study, IL-6 showed promising Se for the detection of marked antrum AG (72%) but with rather poor Sp (41%). Of note, IL-6 values may be influenced by several other conditions (auto-immune diseases, inflammation, physical exercise), and thus this parameter is susceptible to give false-positive results. 

In addition to IL-6, HE-4 turned out to be one of the most promising markers in our study. HE-4 has been mostly investigated in ovarian and endometrial cancer, but several studies have shown that HE-4 expression is increased in GC, particularly of diffuse-type, and its expression correlated with tumor size, stage, and survival [38,39]. More interestingly, HE-4 was upregulated in the metaplastic transition following acute parietal loss cell in mouse and in humans and has been suggested as a surrogate marker of preneoplastic lesions in the stomach [20], such as spasmolytic polypeptide-expressing metaplasia (SPEM) [40]. In the present study, HE-4 appeared of particular interest in combination with PGI/PGII ratio. 

The combination of “functional” (PGI and II) and “morphological” (HE-4) markers could be an interesting approach for studying gastric precancerous lesions in the future.

We confirmed that patients with *H. pylori* infection have increased levels of PGII, probably related to chronic gastric inflammation, and in consequence, they present a lower PGI/PGII ratio, as already reported before [22].

Several points should be taken into account while interpreting the performance of diagnostic markers. First, the performance and usefulness may vary according to the population studied [41,42], the method used [26], the cut-off value set for each parameter [26,43], and the severity of AG [16]. Indeed, in highly selected patients such as in the present study, the prevalence does not reflect the distribution of the disease in the general population. The prevalence of AG varies largely between the Western and the Eastern populations, from 0–8% to more than 80%, respectively [9,44]. Moreover, its distribution varies according to age and ethnicity of the individuals within the same country [44,45,46]. Regarding the tools used to judge the diagnostic performance of a diagnostic test, Se and Sp are the most commonly used. PPV and NPV are also of interest but are influenced by the prevalence of the disease in the studied population, thus limiting the comparison from one study to another. To surmount this limitation, positive and negative likelihood ratios are used. They are expressed as the ratio between the probability of obtaining a positive (or negative) test in sick patients and the probability of obtaining a positive (or negative) test in controls. Usually, a PLR >10 (or NLR <0.1) is considered a sufficient value for assessing the diagnostic, whereas a PLR between 1 and 2 (or NLR 0.5–1) is considered useless.

For the assessment of a biomarker, the cut-off may be adjusted to maximize either Se or Sp. Increasing Se is privileged to exclude the disease (when the test is negative with a high Se) and when a false positive result does not have serious consequences. In the case of AG, this approach could be used in a screening strategy, allowing identification of the patients with positive test and thus those susceptible to bearing GPL. The second approach consists in increasing Sp and could be privileged in the follow up of patients with known GPL, allowing a reduction in the number (and frequency) of follow-up endoscopies. Indeed, systematic endoscopic follow-up of all the patients with GPL is costly, time consuming, not well-accepted, and consequently not well-applied [9]. In several studies, it has been shown that only a small proportion of patients with GPL will develop a GC or progress to more severe lesion [11,27,28,42,47,48,49]. Among these studies, several have shown that *H. pylori* eradication leads to a decreased score of GPL, and even its regression. Thus, one application of non-invasive markers would be to use them regularly to avoid systematic, repeated endoscopies in patients with stable non-invasive marker results. 

Our study has several strengths. The prospective design and the rigorous methodology ensured reliable data. The study was performed under “real-life” conditions, including the data from four different centers, thus allowing generalization of the data for the French population considered as a low GPL prevalence area. This is the first study investigating the new, selected markers suspected to be involved in gastric carcinogenesis, never studied before in this setting. We report here for the first time that IL-6 and HE-4 may be useful for the assessment of antrum AG, and we demonstrate that pepsinogens testing using CLEIA shows good performance for the diagnosis of severe and corpus AG. 

Our study also has some limitations. Only a third of the patients had advanced atrophy, and we did not have enough patients with pangastric advanced atrophy to reliably test the markers in this group. However, the proportion of patients with advanced atrophy was in line with data previously reported in Europe [50,51,52]. A high definition chromoendoscopy, which is known to be superior to white-light endoscopy for the diagnosis of GPL and is currently recommended by the guidelines [13], was not required in the present study. Several studies reported that other factors than the extent of severity of AG could be associated with an increased risk of GC, such as the presence of incomplete type IM [28,29,30,53,54]; however, we were not able to provide these data for our population due to the absence of systematic IM subtyping. We did not perform a cost-efficiency analysis for this study in particular, but a recent and nice review summarized the results of studies conducted in this setting, and addressed the pros and cons in the different situations [55].

In conclusion, this is the first study evaluating PGI and PGII tested by CLEIA, which shows the good diagnostic performance of these markers for the diagnosis of AG in a European population, comparable with previously reported data and comparable with our previous results obtained in the same population with another technique. Additionally, we demonstrate here a potential interest in some new markers, such as HE4 and IL-6 in particular, for the assessment of antrum AG. 

## Figures and Tables

**Table 1:** Serum levels of all the biomarkers in different patient groups according to histology results.

	N	NAG	AGA	AGC	AGAC	*p*-Value
*n* =	113	91	72	42	38	
PG I	70.93 (66.52)	59.81 (44.40)	70.70 (64.52)	14.03 (33.25)	48.45 (51.56)	<0.001
PG II	14.10 (11.52)	13.63 (8.78)	16.56 (16.09)	10.36 (6.08)	13.77 (8.92)	0.027
PGI/PGII	4.86 (1.37)	4.61 (1.75)	4.54 (1.82)	1.07 (1.54)	3.30 (2.68)	<0.001
Adiponectin	5.07 (2.91)	4.31 (2.81)	4.92 (4.10)	5.29 (3.47)	5.31 (3.32)	0.204
Ferritin	91.81 (88.67)	81.22 (61.15)	115.01 (121.68)	68.58 (67.45)	99.95 (98.58)	0.105
HE-4	75.70 (57.59)	73.94 (42.49)	86.42 (49.67)	93.38 (83.34)	115.34 (136.04)	0.012
IL-6	5.28 (11.44)	4.80 (3.83)	4.56 (2.83)	6.86 (11.77)	4.98 (4.62)	0.249
KL-6	291.63 (123.05)	326.02 (181.11)	328.81 (136.57)	353.64 (157.71)	337.21 (197.75)	0.182

N: normal gastric mucosa, NAG: non-atrophic gastritis, AGA: atrophic gastritis of the antrum, AGC: atrophic gastritis of the corpus, AGAC: atrophic gastritis of the antrum and corpus. HE-4: human epididymal protein 4, IL-6: interleukin-6, KL-6: Krebs von den Lungen 6. PGI: pepsinogen I, PGII: pepsinogen II. Results are presented in ng/mL for PGI, PGII, and ferritin; in pg/mL for IL-6; in pmol/l for HE-4; in µg/mL for adiponectin; and in International Units/mL for KL-6.

**Table 2:** Diagnostic performance of different markers for the detection of AG: comparison between all patients with AG (AGA or AGC or AGAC, *n* = 152) and all control patients (N + NAG, *n* = 204), presented for all patients (white space, *n* = 152) and patients with moderate to severe atrophy (grey space, *n* = 54).

	*n* =	AUC	Cut-Off	Se (95%CI)	Sp (95%CI)	PPV (95%CI)	NPV (95%CI)	PLR (95%CI)	NLR (95%CI)
PGI	356	0.642	≤30 *	46.7% (38.6; 55.0)	83.8% (78.0; 88.6)	68.3% (58.4; 77.1)	67.9% (61.7; 73.6)	2.89 (2.02; 4.12)	0.64 (0.54; 0.75)
	356	0.642	≤21.1 #	40.8% (32.9; 49.0)	94.6% (90.6; 97.3)	84.9% (74.6; 92.2)	68.2% (62.4; 73.6)	7.56 (4.13; 13.86)	0.63 (0.55; 0.72)
PGI/PGII	356	0.685	≤3 *	44.7% (36.7; 53.0)	92.6% (88.2; 95.8)	81.9% (72; 89.5)	69.2% (63.4; 74.7)	6.08 (3.62; 10.21)	0.6 (0.51; 0.69)
	356	0.685	≤3.03 #	46.7% (38.6; 55.0)	92.6% (88.2; 95.8)	82.6% (72.9; 89.9)	70.0 % (64.2; 75.4)	6.35 (3.79; 10.64)	0.58 (0.49; 0.67)
Adiponectin	356	0.512	≥6.6	30.3% (23.1; 38.2)	79.4% (73.2; 84.7)	52.3% (41.4; 63.0)	60.4% (54.3; 66.3)	1.47 (1.02; 2.11)	0.88 (0.77; 1.0)
Ferritin	356	0.510	≥150	19.1% (13.2; 26.2)	83.3% (77.5; 88.2)	46.0 % (33.4; 59.1)	58.0 % (52.1; 63.7)	1.14 (0.73; 1.79)	0.97 (0.88; 1.07)
HE4	356	0.606	≥75.8	47.4% (39.2; 55.6)	74.0 % (67.4; 79.9)	57.6% (48.4; 66.4)	65.4% (58.8; 71.5)	1.82 (1.37; 2.43)	0.71 (0.6; 0.84)
IL6	356	0.555	≥4.5	41.4% (33.5; 49.7)	69.1% (62.3; 75.4)	50.0 % (41.0; 59.0)	61.3% (54.7; 67.6)	1.34 (1.02; 1.77)	0.85 (0.72; 1.0)
KL6	356	0.564	≥322	50.7% (42.4; 58.9)	62.3% (55.2; 68.9)	50.0 % (41.8; 58.2)	62.9% (55.8; 69.5)	1.34 (1.06; 1.7)	0.79 (0.65; 0.96)
PGI/PGII +/− HE-4	356	0.687	PGI/PGII ≤ 3.03 OR HE4 ≥ 75.8	69.7% (61.8; 76.9)	67.6% (60.8; 74.0)	61.6% (53.9; 68.9)	75.0 % (68.1; 81.1)	2.16 (1.72; 2.7)	0.45 (0.35; 0.58)
	356	0.614	PGI/PGII ≤ 3.03 AND HE4 ≥ 75.8	23.7% (17.2; 31.3)	99.0% (96.5; 99.9)	94.7% (82.3; 99.4)	63.5% (58.0; 68.8)	24.16 (5.91; 98.78)	0.77 (0.7; 0.84)
PGI	258	0.740	≤30 *	55.6% (41.4; 69.1)	83.8% (78.0; 88.6)	47.6% (34.9; 60.6)	87.7% (82.2; 92.0)	3.43 (2.32; 5.09)	0.53 (0.39; 0.72)
	258	0.740	≤20.2 #	53.7% (39.6; 67.4)	95.6% (91.8; 98.0)	76.3% (59.8; 88.6)	88.6% (83.7; 92.5)	12.17 (6.14; 24.15)	0.48 (0.36; 0.65)
PGI/PGII	258	0.758	≤3 *	55.6% (41.4; 69.1)	92.6% (88.2; 95.8)	66.7% (51.0; 80.0)	88.7% (83.7; 92.6)	7.56 (4.39; 13.0)	0.48 (0.36; 0.65)
	258	0.758	≤3.03	57.4% (43.2; 70.8)	92.6% (88.2; 95.8)	67.4% (52.0; 80.5)	89.2% (84.2; 93.0)	7.81 (4.56; 13.38)	0.46 (0.34; 0.63)
HE-4	258	0.637	≥63.2	70.4% (56.4–82.0)	55.4% (48.3–62.3)	29.5% (21.8–38.1)	87.6% (80.6–92.7)	1.58 (1.25–1.99)	0.53 (0.35–0.82)
PGI/PGII +/− HE-4	258	0.686	PGI/PGII ≤ 3.03 OR HE4 ≥ 63.2	85.2% (72.9; 93.4)	52.0 % (44.9; 59.0)	31.9% (24.4; 40.2)	93.0 % (86.6; 96.9)	1.77 (1.48; 2.12)	0.29 (0.15; 0.55)
		0.684	PGI/PGII ≤3.03 AND HE4 ≥ 63.2	40.7% (27.6; 55.0)	96.1% (92.4; 98.3)	73.3% (54.1; 87.7)	86.0 % (80.8; 90.2)	10.39 (4.9; 22.03)	0.62 (0.49; 0.77)

* Commonly used cut-off, # best cut-off; AUC: area under curve, Se: sensitivity, Sp: specificity, PPV: positive predictive value, NPV: negative predictive value, PLR: positive likelihood ratio, NLR: negative likelihood ratio. N: normal gastric mucosa, NAG: non-atrophic gastritis, AG: atrophic gastritis, AGA: atrophic gastritis of the antrum, AGC: atrophic gastritis of the corpus, AGAC: atrophic gastritis of the antrum and corpus. PGI: pepsinogen I, PGII: pepsinogen II, HE-4: human epididymal protein 4, IL-6: interleukin-6, KL-6: Krebs von den Lungen 6. Results are presented in ng/mL for PGI, PGII and ferritin; in pg/mL for IL-6; in pmol/L for HE-4; in µg/mL for adiponectin; and in International Units/mL for KL-6.

**Table 3:** Diagnostic performance of different markers for the detection of corpus atrophic gastritis: comparison between the patients with AGC + AGAC (*n* = 80) and control patients (N + NAG, *n* = 204), presented for all patients (white space, *n* = 80) and patients with moderate to severe atrophy (grey space, *n* = 36).

	*n* =	AUC	Cut-Off	Se (95%CI)	Sp (95%CI)	PPV (95%CI)	NPV (95%CI)	PLR (95%CI)	NLR (95%CI)
PGI	284	0.782	≤30 *	71.2% (60.0; 80.8)	83.8% (78.0; 88.6)	63.3% (52.5; 73.2)	88.1% (82.7; 92.3)	4.4 (3.13; 6.2)	0.34 (0.24; 0.49)
PGI	284	0.782	≤21.1 #	70.0% (58.7; 79.7)	94.6% (90.6; 97.3)	83.6% (72.5; 91.5)	88.9% (84.0; 92.8)	12.98 (7.18; 23.48)	0.32 (0.23; 0.44)
PGI/PGII	284	0.805	≤3 *	67.5% (56.1; 77.6)	92.6% (88.2; 95.8)	78.3% (66.7; 87.3)	87.9% (82.8; 91.9)	9.18 (5.51; 15.29)	0.35 (0.26; 0.48)
PGI/PGII	284	0.805	≤2.59 #	66.2% (54.8; 76.4)	95.1% (91.2; 97.6)	84.1% (72.7; 92.1)	87.8% (82.7; 91.8)	13.51 (7.24; 25.23)	0.35 (0.26; 0.48)
Adiponectin	284	0.540	≥6.66	37.5% (26.9; 49.0)	79.4% (73.2; 84.7)	41.7% (30.2; 53.9)	76.4% (70.1; 82.0)	1.82 (1.23; 2.69)	0.79 (0.66; 0.95)
Ferritin	284	0.463	≥150	15.0% (8.0; 24.7)	83.3% (77.5; 88.2)	26.1% (14.3; 41.1)	71.4% (65.2; 77.1)	0.9 (0.49; 1.65)	1.02 (0.91; 1.14)
HE-4	284	0.616	≥63.2	67.5% (56.1; 77.6)	55.4% (48.3; 62.3)	37.2% (29.4; 45.7)	81.3% (73.8; 87.4)	1.51 (1.22; 1.88)	0.59 (0.42; 0.82)
IL-6	284	0.549	≥4.2	47.5% (36.2; 59.0)	64.2% (57.2; 70.8)	34.2% (25.5; 43.8)	75.7% (68.6; 81.9)	1.33 (0.99; 1.78)	0.82 (0.65; 1.03)
KL-6	284	0.564	≥421	35.0 % (24.7; 46.5)	85.3% (79.7; 89.9)	48.3% (35.0; 61.8)	77.0 % (70.9; 82.3)	2.38 (1.52; 3.72)	0.76 (0.64; 0.9)
PGI	240	0.856	≤30 *	77.8% (60.8; 89.9)	83.8% (78.0; 88.6)	45.9% (33.1; 59.2)	95.5% (91.4; 98.1)	4.81 (3.36; 6.88)	0.27 (0.14; 0.49)
PGI	240	0.856	≤20.2 #	77.8% (60.8; 89.9)	95.6% (91.8; 98.0)	75.7% (58.8; 88.2)	96.1% (92.4; 98.3)	17.63 (9.09; 34.18)	0.23 (0.13; 0.43)
PGI/PGII	240	0.859	≤3 *	75.0 % (57.8; 87.9)	92.6% (88.2; 95.8)	64.3% (48.0; 78.4)	95.5% (91.5; 97.9)	10.2 (6.05; 17.2)	0.27 (0.15; 0.48)
PGI/PGII	240	0.859	≤0.96 #	72.2% (54.8; 85.8)	98.0 % (95.1; 99.5)	86.7% (69.3; 96.2)	95.2% (91.4; 97.7)	36.83 (13.67; 99.25)	0.28 (0.17; 0.48)

* Commonly used cut-off, # best cut-off; AUC: area under curve, Se: sensitivity, Sp: specificity, PPV: positive predictive value, NPV: negative predictive value, PLR: positive likelihood ratio, NLR: negative likelihood ratio. N: normal gastric mucosa, NAG: non-atrophic gastritis, AGC: atrophic gastritis of the corpus. PGI: pepsinogen I, PGII: pepsinogen II, HE-4: human epididymal protein 4, IL-6: interleukin-6, KL-6: Krebs von den Lungen 6. Results are presented in ng/mL for PGI, PGII and ferritin; in pg/mL for IL-6; in pmol/L for HE-4; in µg/mL for adiponectin; and in International Units/mL for KL-6.

**Table 4:** Diagnostic performance of different markers for the detection of antrum atrophic gastritis: comparison between the patients with AGA (*n* = 72) and control patients (N + NAG, *n* = 204), presented for all patients (white space, *n* = 72) and patients with moderate to severe atrophy (grey space, *n* = 18).

	*n* =	AUC	Cut-off	Se (95%CI)	Sp (95%CI)	PPV (95%CI)	NPV (95%CI)	PLR (95%CI)	NLR (95%CI)
Adiponectin	276	0.520	≤4.22	58.3% (46.1; 69.8)	50.5% (43.4; 57.5)	29.4% (22.1; 37.6)	77.4% (69.4; 84.2)	1.18 (0.93; 1.5)	0.83 (0.61; 1.12)
Ferritin	276	0.563	≥150	23.6% (14.4; 35.1)	83.3% (77.5; 88.2)	33.3% (20.8; 47.9)	75.6% (69.4; 81.0)	1.42 (0.85; 2.37)	0.92 (0.8; 1.06)
HE-4	276	0.595	≥77.6	45.8% (34.0; 58.0)	74.5% (68.0; 80.3)	38.8% (28.4; 50.0)	79.6% (73.2; 85.1)	1.8 (1.28; 2.54)	0.73 (0.58; 0.91)
IL-6	276	0.561	≥5.1	36.1% (25.1; 48.3)	77.0 % (70.6; 82.6)	35.6% (24.7; 47.7)	77.3% (71.0; 82.9)	1.57 (1.05; 2.33)	0.83 (0.69; 1.0)
KL-6	276	0.564	≥226	77.8% (66.4; 86.7)	33.8% (27.4; 40.8)	29.3% (23.0; 36.3)	81.2% (71.2; 88.8)	1.18 (1.0; 1.38)	0.66 (0.41; 1.05)
Adiponectin	258	0.501	≥8.47	22.2% (6.4; 47.6)	88.2% (83.0; 92.3)	14.3% (4.0; 32.7)	92.8% (88.2; 96.0)	1.89 (0.74; 4.85)	0.88 (0.69; 1.13)
Ferritin	258	0.550	≥150	16.7% (3.6; 41.4)	83.3% (77.5; 88.2)	8.1% (1.7; 21.9)	91.9% (87.0; 95.4)	1.0 (0.34; 2.94)	1.0 (0.81; 1.24)
HE-4	258	0.600	≥64.8	66.7% (41.0; 86.7)	56.9% (49.8; 63.8)	12.0 % (6.4; 20.0)	95.1% (89.6; 98.2)	1.55 (1.08; 2.22)	0.59 (0.3; 1.14)
IL-6	258	0.588	≥3.1	72.2% (46.5; 90.3)	41.2% (34.4; 48.3)	9.8% (5.3; 16.1)	94.4% (87.4; 98.2)	1.23 (0.9; 1.67)	0.67 (0.31; 1.45)
KL6	258	0.565	≥192	94.4% (72.7; 99.9)	22.5% (17.0; 28.9)	9.7% (5.8; 15.1)	97.9% (88.7; 99.9)	1.22 (1.07; 1.39)	0.25 (0.04; 1.68)

AUC: area under curve, Se: sensitivity, Sp: specificity, PPV: positive predictive value, NPV: negative predictive value, PLR: positive likelihood ratio, NLR: negative likelihood ratio. N: normal gastric mucosa, NAG: non-atrophic gastritis, AGA: atrophic gastritis of the antrum. HE-4: human epididymal protein 4, IL-6: interleukin-6, KL-6: Krebs von den Lungen 6. Results are presented in ng/mL for ferritin, in pg/mL for IL-6, in pmol/l for HE-4, in µg/mL for adiponectin, and in International Units/mL for KL-6.

**Table 5:** Diagnostic performance of different markers for the detection of pangastric (antrum and corpus) atrophic gastritis: comparison between the patients with AGAC (*n* = 38) and control patients (N + NAG, *n* = 204).

	*n*	AUC	Cut-Off	Se (95%CI)	Sp (95%CI)	PPV (95%CI)	NPV (95%CI)	PLR (95%CI)	NLR (95%CI)
PGI	242	0.613	≤30 *	47.4% (31.0; 64.2)	83.8% (78.0; 88.6)	35.3% (22.4; 49.9)	89.5% (84.3; 93.5)	2.93 (1.85; 4.63)	0.63 (0.46; 0.85)
PGI	242	0.613	≤21.1 #	47.4% (31.0; 64.2)	94.6% (90.6; 97.3)	62.1% (42.3; 79.3)	90.6% (85.9; 94.2)	8.78 (4.52; 17.09)	0.56 (0.41; 0.75)
PGI/PGII	242	0.664	≤3 *	44.7% (28.6; 61.7)	92.6% (88.2; 95.8)	53.1% (34.7; 70.9)	90.0% (85.1; 93.7)	6.08 (3.33; 11.11)	0.6 (0.45; 0.8)
PGI/PGII	242	0.664	≤2.86 #	44.7% (28.6; 61.7)	92.6% (88.2; 95.8)	53.1% (34.7; 70.9)	90.0% (85.1; 93.7)	6.08 (3.33; 11.11)	0.6 (0.45; 0.8)
Adiponectin	242	0.542	≥6.79	44.7% (28.6; 61.7)	79.9% (73.7; 85.2)	29.3% (18.1; 42.7)	88.6% (83.1; 92.8)	2.23 (1.42; 3.48)	0.69 (0.52; 0.93)
Ferritin	242	0.527	≥150	21.1% (9.6; 37.3)	83.3% (77.5; 88.2)	19.0% (8.6; 34.1)	85.0% (79.3; 89.6)	1.26 (0.63; 2.51)	0.95 (0.8; 1.13)
HE-4	242	0.638	≥75.8	52.6% (35.8; 69.0)	74.0% (67.4; 79.9)	27.4% (17.6; 39.1)	89.3% (83.7; 93.6)	2.03 (1.38; 2.96)	0.64 (0.45; 0.9)
IL-6	242	0.529	≥6.4	31.6% (17.5–48.7)	83.8% (78.0; 88.6)	26.7% (14.6–41.9)	86.8% (81.3–91.2)	1.95 (1.11–3.43)	0.82 (0.65–1.02)
KL-6	242	0.525	≥400	36.8% (21.8; 54.0)	80.4% (74.3; 85.6)	25.9% (15.0; 39.7)	87.2% (81.6; 91.6)	1.88 (1.14; 3.1)	0.79 (0.61; 1.01)

* Commonly used cut-off, # best cut-off; AUC: area under curve, Se: sensitivity, Sp: specificity, PPV: positive predictive value, NPV: negative predictive value, PLR: positive likelihood ratio, NLR: negative likelihood ratio. N: normal gastric mucosa, NAG: non-atrophic gastritis, AGAC: atrophic gastritis of the antrum and corpus. HE-4: human epididymal protein 4, IL-6: interleukin-6, KL-6: Krebs von den Lungen 6. Results are presented in ng/mL for ferritin, in pg/mL for IL-6, in pmol/L for HE-4, in µg/mL for adiponectin, and in International Units/mL for KL-6.

## Data Availability

All raw data are available upon request.

## Supplementary Materials

The following supporting information can be downloaded at: https://www.mdpi.com/article/10.3390/diagnostics12030695/s1, Table S1: Post hoc analysis (Tukey’s test) of the comparison between the different histological subgroups (only the markers for which the significant differences were found are presented), Table S2: Diagnostic performance of different biomarkers for the detection of atrophic gastritis: comparison between the control patients (N+NAG, *n* = 164) and patients with atrophic gastritis (AGA + AGC + AGAC, *n* = 119) without PPI treatment, Table S3: Comparison of diagnostic performance of PG I (A) and PGI/PGII (B) testing for the detection of any atrophic gastritis (AG) and corpus atrophic gastritis (AGC+ AGAC) between the current study (Fujirebio® test) and previous study (Gastropanel). Comparison of the ROC curves using the DeLong test.
