# Supplementary material for: Serum Pepsinogens Combined with New Biomarkers Testing Using Chemiluminescent Enzyme Immunoassay for Non-Invasive Diagnosis of Atrophic Gastritis: A Prospective, Multicenter Study
Source: Diagnostics (Basel). 2022 Mar 12;12(3):695. doi: 10.3390/diagnostics12030695 (PMC8947400; doi:10.3390/diagnostics12030695)
Supplement: Supplementary file 1 [file diagnostics-12-00695-s001.zip › diagnostics-1599853-supplementary.pdf]

Supplementary materials.

Article: Serum pepsinogens combined with new biomarkers testing using Chemiluminescent Enzyme Immunoassay for non-invasive diagnosis of atrophic gastritis: A prospective, multicenter study

Journal name: Gastric cancer

Author names: Nicolas Chapelle, Malgorzata Osmola, Jérôme Martin, Justine Blin, Maxime Leroy, Iva Jirka, Driffa Moussata, Dominique Lamarque, Raphael Olivier, David Tougeron, Anne Hay-Lombardie, Edith Bigot-Corbel, Damien Masson, Jean-François Mosnier, Tamara Matysiak-Budnik

Corresponding author: Pr. Tamara Matysiak Budnik

Email: tamara.matysiakbudnik@chu-nantes.fr

Supplementary Table S1. Post-hoc analysis (Tukey's test) of the comparison between the different histological subgroups (only the markers for which the significant differences were found are presented)

	HE-4		PGI		PGII		PGI/PGII ratio	
	**Difference [95%CI]**	**adjusted p-value**	**Difference [95%CI]**	**adjusted p-value**	**Difference [95%CI]**	**adjusted p-value**	**Difference [95%CI]**	**adjusted p-value**
N-NAG	-1.8 [-28.5 ; 25.0]	0.999	-11.1 [-32.9 ; 10.6]	0.626	-0.5 [-4.8 ; 3.9]	0.998	-0.3 [-0.9 ; 0.4]	0.847
N-AGA	-10.7 [-39.3 ; 17.9]	0.842	0.2 [-23.0 ; 23.5]	0.999	-2.5 [-7.1 ; 2.2]	0.600	0.3 [-0.4 ; 1.0]	0.756
N-AGC	-17.7 [-52.0 ; 16.6]	0.618	56.9 [29.0 ; 84.8]	**<0.001**	3.7 [-1.8 ; 9.3]	0.355	3.8 [2.9 ; 4.7]	**<0.001**
N-AGAC	-39.6 [-75.2 ; -4.1]	**0.020**	22.5 [-6.5 ; 51.4]	0.210	0.23 [-5.5 ; 6.1]	0.999	1.6 [0.7 ; 2.5]	**< 0.001**

NAG-AGA	-12.5 [-42.4 ; 17.4]	0.782	-10.9 [-35.2 ; 13.5]	0.736	-2.9 [-7.8 ; 1.95]	0.468	0.1 [-0.7 ; 0.8]	0.999
NAG-AGC	-19.4 [-54.8 ;15.9]	0.526	45.8 [17.0 ; 74.6]	**<0.001**	3.3 [-2.5 ; 9.0]	0.531	3.5 [2.6 ; 4.4]	**<0.001**
NAG-AGAC	-41.4 [-78.0 ; -4.8]	**0.011**	11.4 [-18.5 ; 41.2]	0.834	-0.1 [-6.1 ; 5.8]	0.999	1.3 [0.4 ; 2.2]	**0.001**
AGC-AGA	7.0 [-29.9 ; 43.8]	0.986	-56.7 [-86.6 - -26.7]	**<0.001**	-6.2 [-12.20 ; -0.19]	**0.039**	-3.5 [-4.4 ; -2.5]	**<0.001**
AGC-AGAC	-22.0 [-64.4 ; 20.5]	0.616	-34.4 [-69.0 ; 0.1]	0.051	-3.4 [-10-3 ; 3.52]	0.662	-2.2 [-3.3 ; -1.2]	**< 0.001**
AGAC-AGA	29.9 [-9.1 ; 66.9]	0.228	-22.2 [-53.2 ; 8.7]	0.283	-2.8 [-9.0 ; 3.4]	0.731	-1.2 [-2.2 ; -0.3]	**0.004**

N: normal gastric mucosa, NAG: non-atrophic gastritis, AGC: atrophic gastritis of the corpus, AGA: atrophic gastritis of the antrum, AGAC: atrophic gastritis of the antrum and corpus, PGI: Pepsinogen I, PGII: Pepsinogen II, HE-4: human epididymal protein 4, Results are presented in ng/ml for PGI and PGII, and in pmol/l for HE-4.

Supplementary Table S2. Diagnostic performances of different biomarkers for the detection of atrophic gastritis: comparison between the control patients (N+NAG, n= 164) and patients with atrophic gastritis (AGA + AGC + AGAC, n= 119) without PPI treatment

	n=	AUC	Cut-off	Se [95%CI]	Sp [95%CI]	PPV [95%CI]	NPV [95%CI]	PLR [95%CI]	NLR [95%CI]
PGI	283	0,661	≤ 30*	51.3 % [41.9 ; 60.5]	81.1 % [74.3 ; 86.8]	66.3 % [55.7 ; 75.8]	69.6 % [62.6 ; 76.1]	2.71 [1.89 ; 3.9]	0.60 [0.49 ; 0.73]
PGI	283	0,661	≤ 21.1#	44.5 % [35.4 ; 53.9]	93.9 % [89.1 ; 97.0]	84.1 % [72.7 ; 92.1]	70.0 % [63.5 ; 76.0]	7.3 [3.88 ; 13.76]	0.59 [0.50 ; 0.70]
PGI/PGII	283	0,697	≤ 3*	47.9 % [38.7 ; 57.2]	92.1 % [86.8 ; 95.7]	81.4 % [70.3 ; 89.7]	70.9 % [64.3 ; 76.9]	6.04 [3.47 ; 10.52]	0.57 [0.47 ; 0.68]
PGI/PGII	283	0,697	≤ 3.03#	49.6 % [40.3 ; 58.9]	92.1 % [86.8 ; 95.7]	81.9 % [71.1 ; 90.0]	71.6 % [65.0 ; 77.5]	6.25 [3.6 ; 10.86]	0.55 [0.46 ; 0.66]
Adiponectin	283	0,524	≥ 5.49	37.8 % [29.1 ; 47.2]	72.6 % [65.1 ; 79.2]	50.0 % [39.3 ; 60.7]	61.7 % [54.4 ; 68.5]	1.38 [0.98 ; 1.93]	0.86 [0.72 ; 1.01]
Ferritin	283	0,521	≥ 150	18.5 % [12.0 ; 26.6]	82.3 % [75.6 ; 87.8]	43.1 % [29.3 ; 57.8]	58.2 % [51.6 ; 64.6]	1.05 [0.63 ; 1.73]	0.99 [0.89 ; 1.11]
HE-4	283	0,600	≥ 59.2	68.9 % [59.8 ; 77.1]	53.7 % [45.7 ; 61.5]	51.9 % [43.8 ; 59.9]	70.4 % [61.6 ; 78.2]	1.49 [1.21 ; 1.82]	0.58 [0.43 ; 0.78]
IL-6	283	0,570	≥ 4.2	47.1 % [37.8 ; 56.4]	65.9 % [58.1 ; 73.1]	50.0 % [40.4 ; 59.6]	63.2 % [55.5 ; 70.4]	1.38 [1.04 ; 1.83]	0.80 [0.66 ; 0.98]
KL-6	283	0,566	≥ 322	49.6 % [40.3 ; 58.9]	64.0 % [56.2 ; 71.4]	50.0 % [40.7 ; 59.3]	63.6 % [55.8 ; 71.0]	1.38 [1.05 ; 1.81]	0.79 [0.64 ; 0.97]

*commonly used cut-off, #best cut-off, AUC: area under curve, Se: sensitivity, Sp: specificity, PPV: positive predictive value, NPV: negative predictive value, PLR: positive likelihood ratio, NLR: negative likelihood ratio. N: normal gastric mucosa, NAG: non-atrophic gastritis, AGA: atrophic

gastritis of the antrum, AGC: atrophic gastritis of the corpus, AGAC: atrophic gastritis of the antrum and corpus. PGI : Pepsinogen I, PGII: Pepsinogen II, HE-4: human epididymal protein 4, IL-6: Interleukin-6, KL-6: Krebs von den Lungen 6. Results are presented in ng/ml for PGI, PGII and ferritin, in pg/ml for IL-6, in pmol/l for HE-4, in µg/ml for adiponectin, and in International Units/ml for KL-6.

Supplementary Table S3: Comparison of diagnostic performances of PG I (A) and PGI/PGII (B) testing for the detection of any atrophic gastritis (AG) and corpus atrophic gastritis (AGC+ AGAC) between the current study (Fujirebio® test) and previous study (Gastropanel). Comparison of the ROC curves using the DeLong test.

A. PG I

Population	GASTRO-PRA AUC [95% CI]	Fujirebio	p-value
AG	0.679 [0.619 – 0.738]	0.642 [0.580 – 0.704]	0.776
AGC+ AGAC	0.792 [0.716 – 0.867]	0.782 [0.706 – 0.859]	0.866

B. PGI/PGII ratio

Population	GASTRO-PRA AUC [95% CI]	Fujirebio	p-value
AG	0.679 [0.619 – 0.738]	0.685 [0.625 – 0.745]	0.882
AGC + AGAC	0.815 [0.747 – 0.883]	0.805 [0.736 – 0.874]	0.842
